# Natural orifice transluminal endoscopic surgery for colorectal cancer

**DOI:** 10.1002/bjs5.4

**Published:** 2017-05-24

**Authors:** P. N. Hiep, H. H. Thien, P. A. Vu, P. H. Thanh, N. T. Xuan

**Affiliations:** ^1^ Department of Paediatric and Abdominal Emergency Surgery Hue Central Hospital Hue Vietnam; ^2^ Hue University of Medicine and Pharmacy Hue Vietnam

## Abstract

**Background:**

Natural orifice transluminal endoscopic surgery (NOTES) has theoretical advantages over other approaches.

**Methods:**

This was a prospective cohort study of colorectal cancers operated on by NOTES (transanally for rectal tumours, transvaginally for sigmoid tumours) between December 2013 and December 2015, with a minimum follow‐up of 1 year. Eligibility criteria included ASA fitness grade I–III, BMI below 25 kg/m^2^ and TNM stage T3 N0 M0. Exclusion criteria included pregnancy or distant metastasis. The anastomosis was either handsewn or performed mechanically.

**Results:**

Sixteen patients were operated on by a transanal and four by a transvaginal approach. There were ten men and ten women, with a mean(s.d.) age of 55·6(12·1) years. Mean BMI was 22·4(2·6) kg/m^2^. Four anterior, 11 low anterior and five intersphincteric resections were performed for 16 rectal and four low sigmoid tumours. The mean duration of surgery was 258(11) min. No conversion to laparotomy was needed, and there were no deaths. Five patients required additional ports, for intraoperative bleeding (1), suture of an intraoperative urethral injury with covering ileostomy (1) and difficulty in dissection (3). One patient had an anastomotic leak requiring transanal closure and ileostomy on day 7. Both ileostomies were closed after 2 months. The mean hospital stay was 6·4(1·8) days. All resections were R0.

**Conclusion:**

In carefully selected patients NOTES for colorectal cancer resection was feasible and effective.

## Introduction

Since the first transgastric liver biopsy by Kalloo and colleagues^1^, there has been continued interest in natural orifice transluminal endoscopic surgery (NOTES)^2^, ^3^, ^4^, ^5^, ^6^, ^7^, ^8^, ^9^, ^10^, ^11^, ^12^, ^13^, ^14^. For colorectal cancer, most operations have been hybrid procedures^4^, ^5^, ^6^, ^7^, ^8^, ^9^, ^10^ with few reports^11^, ^12^, ^13^, ^14^ confined to NOTES alone. In colorectal cancer surgery, potential benefits of NOTES might include less pain, avoidance of incisional infection, shorter hospital stay, better cosmesis and high‐quality oncological resection by transanal total mesorectal excision (TME)^4^, ^5^, ^6^, ^7^, ^8^, ^9^, ^10^, ^11^, ^12^, ^13^, ^14^.

The aim of this study was to report a technique, and early results, for transanal and transvaginal colorectal resection of colorectal cancer at a single centre.

## Methods

Selected patients with colorectal cancer who gave informed consent for rectal or sigmoid resection via a NOTES technique were included. All underwent operation at Hue Central Hospital, one of the three largest polyclinic hospitals in Vietnam, with 2400 beds, responsible for almost all patients with cancer in the central region of the country. Hospital ethics committee approval was obtained for this cohort study, with funding provided by the National Scientific Programme of the Vietnamese Ministry of Science and Technology, on condition of assessment of outcomes at the end of the third year, meaning operations concluded within 2 years.

Sigmoid colon was defined as colon more than 12 cm from the anal verge by rigid proctoscopy^15^. The rectum was divided into three parts: lower (3–6 cm from anal verge), middle (more than 6 to 9 cm) and upper (more than 9 to 12 cm)^16^.

Patient selection criteria included: ASA fitness grade I–III, not overweight (BMI below 25 kg/m^2^)^17^ ^18^, tumour size less than 5 cm (length of tumour based on MRI), and preoperative TNM stage T3 N0 M0 or less^19^, based on MRI, abdominal CT and chest X‐ray. No patient had intestinal obstruction. Only postmenopausal women with sigmoid cancer, without inflammatory or infectious vaginal disease, were considered for transvaginal endoscopic surgery. Patients considered to have N1 disease received short‐course radiotherapy and were reassessed after 6 weeks by MRI and endoultrasonography.

### Surgical technique

Preoperative preparation was similar to that for conventional laparoscopic colorectal resection. Under general anaesthesia, the patient was placed in the lithotomy position with insertion of a bladder catheter. Both the surgeon and first assistant stood between the patient's legs. The laparoscopic tower was placed on the patient's left. A SILS™ Port multiple access port (Covidien, Minneapolis, Minnesota, USA) was used along with a 30° telescope (5·5 mm, 50 cm) and standard laparoscopic graspers of different lengths.

**Figure 1:**
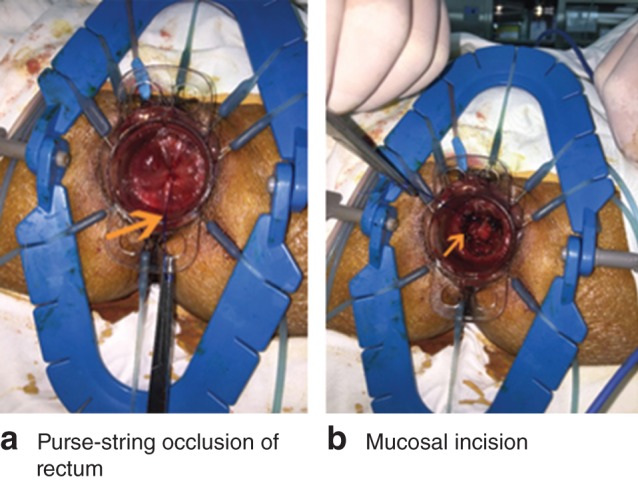
Mucosal dissection. **a** Purse‐string occlusion of the rectum (arrow) and **b** mucosal incision (arrow). Traction on the purse‐string facilitates the mucosal incision

**Figure 2:**
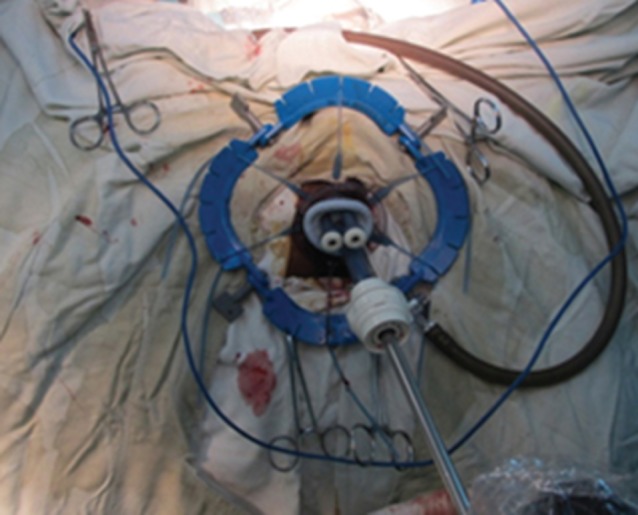
SILS™ Port inserted into anus

**Figure 3:**
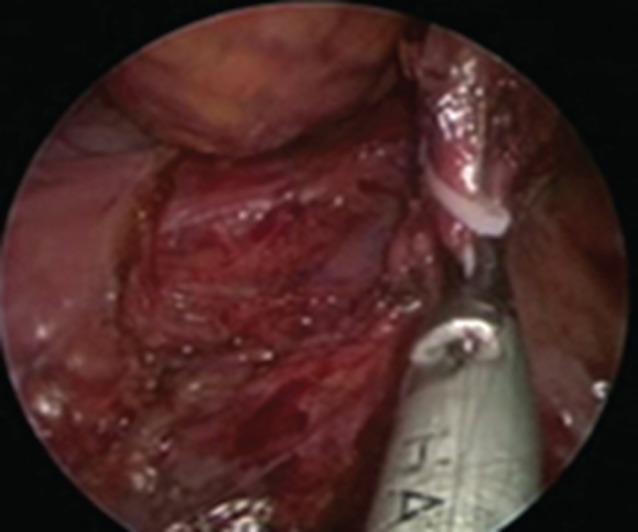
Inferior mesenteric artery ligated with a Hem‐o‐lok^®^ clip

For the transanal approach, a Lone Star^®^ retractor (CooperSurgical, Trumbull, Connecticut, USA) (for low rectal cancer) alone or combined with a Covidien haemorrhoidectomy anal dilator (for middle and high rectal tumours) was placed around or in the anus. The rectal lumen was disinfected before and after closure. The rectal lumen was closed with a Prolene^®^ (Ethicon, Cornelia, Georgia, USA) 2/0 purse‐string suture, 1 cm below the distal margin of the tumour. Mucosal dissection started another 1 cm below this point (*Fig*. 1), posteriorly, with monopolar electrocautery and completed circumferentially. The SILS™ Port was placed when the space created was large enough (*Fig*. 2). Carbon dioxide insufflation was maintained at 12 mmHg. TME was continued with a harmonic scalpel or monopolar hook. The peritoneal fold was opened anteriorly and then laterally. The rectum was then pushed into the abdominal cavity. The inferior mesenteric artery (IMA) and vein (IMV) were divided either with Hem‐o‐lok® clips (Weck Closure Systems, Research Triangle Park, North Carolina, USA) (*Fig*. 3) or a vascular Endo GIA™ stapler (Covidien). The Toldt fascia was freed until an adequate colonic length for the future pull‐through had been obtained. To protect the non‐peritoneal area and distal rectal stump, a nylon bag was inserted into the abdominal cavity through the anus, and the tumour‐bearing intestinal segment was then pulled out through its lumen and resection performed at least 6 cm proximal to the tumour. The anastomosis was handsewn if the distal stump was less than or equal to 2 cm from the dentate line, or performed using a circular stapler (EEA^™^ device; Covidien) if the distal stump was longer than 2 cm.

**Figure 4:**
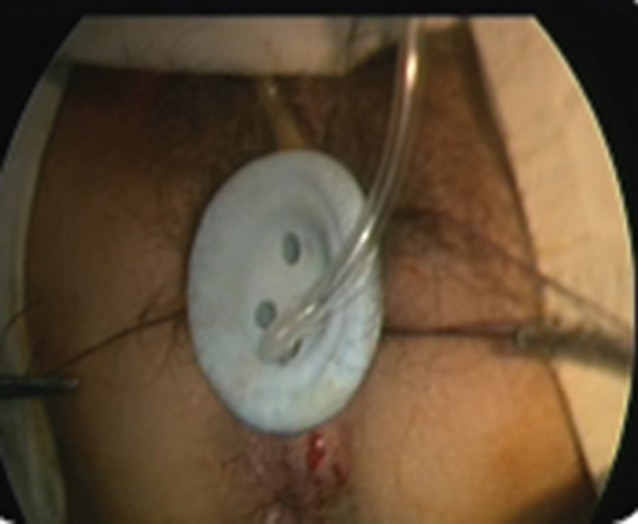
SILS™ Port inserted into vagina

For the transvaginal approach, the posterior fornix was opened between stay sutures over approximately 2·5 cm, and the SILS™ Port device placed (*Fig*. 4). The sigmoid was divided 2 cm distal to the tumour through a mesenteric window created close to the sigmoid. The IMA and IMV were divided either with Hem‐o‐lok® clips or using the vascular Endo GIA™ device. The Toldt fascia was freed to obtain adequate colonic length for the future pull‐through. The opening of the vaginal fornix was protected with a nylon bag and the tumour pulled out through its lumen. The colon was resected at least 6 cm proximal to the tumour and prepared for anastomosis. The anastomosis was performed with a circular stapler under direct vision through the posterior vaginal fornix.

Additional 5 mm port(s), transumbilically, in the right lower quadrant and/or left lower quadrant, were used as needed, and in this order of priority, in case of intraoperative difficulties.

### Postoperative assessment and analysis

Type of resection and anastomosis, duration of operation, conversion to traditional laparoscopy or laparotomy, need for additional port(s), intraoperative events and postoperative complications were noted. Postoperative pain was evaluated by means of a visual analogue scale (VAS). The standard pain therapy was intravenous paracetamol (60 mg per kg per 24 h), given after pain evaluation.

Specimen length, Quirke's grading^20^ for TME, TNM stage^19^, and sphincter function according to Horgan and colleagues^21^ were recorded. Those with postoperative stage III histology received adjuvant therapy. Follow‐up included clinical examination, carcinoembryonic antigen measurement, colonoscopy and abdominal CT.

Patient data are shown as mean(s.d.) unless indicated otherwise.

## Results

Among a total of 92 patients with colonic cancer and 84 with rectal cancer, 20 were selected to undergo elective surgery for rectal or low sigmoid cancer between December 2013 and December 2015. Of these, 16 had transanal resections for rectal cancer and four had transvaginal resections for sigmoid cancer. Follow‐up ended in November 2016. There were ten men and ten women of mean age 55·6(12·1) years. BMI was 22·4(2·6) kg/m^2^. Preoperative tumour characteristics are shown in *Table* 1.

**Table 1:** Preoperative tumour characteristics

	T2	T3
Rectum		
Low	1	5
Middle	3	3*
High	4	0
Sigmoid	4	0
Total	12	8

* One patient with a mid‐rectal tumour and suspected lymph node invasion on MRI (T3 N1 M0) received a short course of neoadjuvant radiotherapy 4 weeks before operation. Preoperative stage based on MRI was T3 N0 M0 and pTNM stage was T3 N0 M0.

There were four anterior resections for the low sigmoid tumours, 11 low anterior resections and five intersphincteric resections. Intestinal continuity was restored by circular stapling (14) or handsewn anastomosis (6). Mean duration of operation was 258(11) min. There were no conversions to laparotomy. Five patients required additional ports. One patient with intraoperative bleeding was managed successfully by two additional ports placed in the abdomen. One intraoperative urethral injury required an additional port to suture the urethra and perform a temporary ileostomy. The reasons for all additional ports are detailed in *Table* 2. There were no deaths.

**Table 2:** Reasons for additional ports

	Approach	No. of trocars	No. of patients
Haemorrhage	Transanal	2	1
Limited working space	Transanal	1	1
Disorientation	Transanal	1	1
Thick mesocolon*	Transvaginal	2	1
Urethral injury	Transanal	1	1

* BMI 27·3 kg/m^2^.

The mean interval before return of bowel movements was 2(1) days. The mean hospital stay was 6·4(1·8) days. The mean VAS for pain evaluation on the first day after surgery was 3·4(0·5) points. A single anastomotic leak following an intersphincteric resection was dealt with by transanal closure and ileostomy on postoperative day 7. The patient had normal defaecation at 3 months after ileostomy closure. Both patients with ileostomies made otherwise uneventful recoveries and the stomas were closed after 2 months.

All resections were classed as R0. The definitive pTNM stage^19^ is shown in *Table* 3. The mean length of resected specimens was 28·4(4·5) cm. Quirke's assessment for TME^20^ was good (grade 3) for all 16 rectal cancers. Sphincter function was graded 1 in all patients at 3 months^21^.

**Table 3:** Pathological TNM stage

	Tumour stage			
	I	II	III	
TNM	T2 N0 M0	T3 N0 M0	T2 N1 M0	T3 N1 M0
Rectum	3	5	5	3
Sigmoid	4	0	0	0

During follow‐up, one patient developed an anastomotic stenosis 3 months after low anterior resection that was managed successfully by anal dilatation. No evidence of recurrence was detected by abdominal CT and colonoscopy at 6 months and 1 year. After a median follow‐up of 16 months, all patients remained alive and recurrence‐free.

## Discussion

Transanal and transvaginal NOTES procedures were feasible in this selected population. The mean operating time of 4 h and 18 min, and rates of complications seem reasonable.

Laparoscopic surgery has become increasingly popular in surgical practice and in the treatment of colorectal cancer^22^ ^23^. The first report^8^ of transanal rectal resection in humans in 2010 described a transanal TME in a 76‐year‐old woman with a T2 N2 rectal cancer treated with preoperative chemoradiation. The procedure was aided by laparoscopic visualization, retraction and exposure through one 5‐mm port (later used as the stoma site) and 2‐mm needle ports (1 of which was used as a drain site). The specimen was transected transanally followed by handsewn coloanal anastomosis.

Laparoscopic natural orifice specimen extraction for very low rectal cancer began at the present centre in 2007, with subsequent technical development^24^ ^25^. For women with low sigmoid cancer, a transvaginal NOTES procedure was adopted; this seems to have been described previously only for benign disease^26^.

With regard to surgical and oncological safety, careful selection, technical competence, and satisfactory short‐ and long‐term outcomes are required. In this study, only patients with tumour stage T3 or less and N0 status were considered. Obesity according to Asian standards (BMI over 25 kg/m^2^)^21^ ^22^ was also seen as a contraindication, although one patient in the present series had a BMI of 27·3 kg/m^2^. Whether the complete NOTES approach to TME will be ideal in the narrow pelvis of an obese man needs further evaluation^27^, ^28^, ^29^, ^30^. In the largest series to date looking at transanal TME^30^, more than half of the patients had a BMI greater than 25 kg/m^2^ and 12 per cent had a BMI over 30 kg/m^2^ without any particular adverse influence on outcome.

Additional ports were required for various reasons in five of 20 patients in the present study. In one of these patients, this was due to intraoperative bleeding, which was controlled after placing two additional trocars in the abdomen. Massive bleeding also occurred in one patient in the series of Kang *et al*.^13^. In one other patient, previous radiotherapy made the dissection difficult and further ports were placed to clarify the dissection planes.

There was also a single intraoperative urethral injury, which was small and 6 cm from the anal verge. The injury was sutured transanally after removing the SILS™ Port. Ileostomy was performed to eliminate the risk of any intestinal leak compromising healing of the urethra. This type of injury appears as an individual event in other observational series of transanal operations^13^ ^27^, ^28^.

One theoretical problem related to NOTES is the risk of intra‐abdominal cavity infection due to the colon being pulled out through the anus or vagina. As in the present series, other reports^4^, ^5^, ^6^, ^7^, ^8^, ^9^, ^10^, ^11^ ^24^, ^25^ have described the absence of intra‐abdominal infection.

Most publications on NOTES have concentrated on short‐term outcomes^11^, ^12^, ^13^, ^14^. Among studies of colorectal cancer treated with hybrid NOTES, no local recurrence has been reported at 6 months^6^ ^24^, ^25^ or 9 months^11^. There was a single local recurrence in one series^31^ with a median follow‐up of 29 months. The present study had satisfactory oncological results at a median follow‐up time of 16 months, reflecting the fact that all patients had an R0 resection, with good (stage 3) specimen quality according to Quirke's TME assessment^20^ in all rectal resections.

All 20 patients in this study considered that they had perfect continence at 3 months, although it is acknowledged that this short‐term assessment of sphincter function is a limitation in the present study.

Transanal and transvaginal NOTES in selected patients with colorectal cancer seems feasible and effective. Clear indications for this procedure need to be defined and its safety merits further study.
